# A Network Pharmacology Based Research on the Mechanism of Donepezil in Treating Alzheimer's Disease

**DOI:** 10.3389/fnagi.2022.822480

**Published:** 2022-04-08

**Authors:** Lihua Liu, Yingying Zhu, Peng Fu, Jundong Yang

**Affiliations:** ^1^Laizhou City People's Hospital, Laizhou, Yantai, China; ^2^Department of Pharmacy, Shanghai Changhai Hospital, Naval Medical University, Shanghai, China

**Keywords:** donepezil, Alzheimer's disease, network pharmacolgy, mechanism, prognosis

## Abstract

**Objective:**

In order to explore and further understand the efficacy of donepezil (DNP) in the treatment of Alzheimer's disease (AD), this research was conducted based on network pharmacology and molecular docking.

**Method:**

Compounds of DNP and its effective targets were collected using the TCMSP Chinese medicine system pharmacology database. Disease targets were screened and selected utilizing GeneCards, TTD, DrugBank, CTD, and other online databases. Then, Venn diagrams were generated to identify the intersections. A diseases-drug-active ingredient-key target protein interaction (PPI) network was constructed using the STING database. GO and KEGG enrichment analyses were conducted to predict the function and mechanism of DNP, which were visualized by graphs and bubble charts. After the screening, the top five interacting targets in the PPI network and the compound containing the most active target were selected for molecular docking.

**Results:**

The study received 110 potential targeting genes and 155 signaling pathways. A strong association between DNP and modulation of chemical synaptic transmission and the regulation of trans-synaptic signaling is noted. Signaling pathways related to the proliferation, differentiation, and survival of cells are also found positively relative. The results revealed that the mechanism of its therapeutic effect is multi-component, multi-target, and multi-pathway, laying a foundation for the follow-up in-depth study of the mechanism of DNP in the treatment of AD.

**Conclusion:**

This research provides a superior prediction that AD could be treated using DNP which targets the key proteins and essential pathways associated with the recovery of AD.

## Introduction

Alzheimer's disease (AD) is a neurodegenerative disorder in the central nervous system, prevalently observed among the elderly and near-elderly, characterized by progressive cognitive impairment, and memory degeneration (Hebert et al., 1995; Alzheimer's, 2016).

Credited as the most common cause of dementia, AD is known for its patients suffering from a long course of the disease, loss of self-care ability, and eventual death from comorbidities, identified by the World Health Organization as a prior global public health concern (Barker et al., 2002; Wilson et al., 2012).

In 2020, an estimated number of AD cases worldwide was reported to be 24 million, which was predicted to increase fourfold by the year 2050 (Breijyeh and Karaman, 2020).

Since the first AD case was reported in 1907 by Alois Alzheimer, significant gains in our understanding of the etiology and pathogenesis of AD have been achieved. Nevertheless, current therapeutic strategies for AD remain considerably limited, with only two alternatives available, including naturally derived and synthetic inhibitors or hybrid analogs to cholinesterase enzyme, and antagonists to N-methyl D-aspartate (NMDA). Despite the effectiveness of the existing therapies in treating the symptoms of AD, no significant influence has been found in curing or preventing the disease (Wang and Reddy, 2017). Acetylcholinesterase inhibitors (AChEIs) are categorized as the former, blocking cholinesterase enzymes from degrading ACh to increase ACh levels in the synaptic cleft. For patients with all levels of AD, whether mild, moderate, or severe (Winblad et al., 2001), donepezil (DNP), an AChEI, is wildly commended in China as the most promising therapeutic candidate for AD (Deardorff and Grossberg, 2016). Studies have confirmed the efficacy and safety of DNP for patients with mild to moderate AD and its possible effectiveness for severe AD (Zhang and Gordon, 2018). However, it should also be noted that DNP is likewise only capable of treating the symptoms of AD but cannot reverse or alter the AD progression (Cacabelos, 2007).

The insufficient understanding of the pathogenesis of AD sets great obstacles in the path of finding a novel breakthrough in the improvement of the existing drugs. Therefore, the investigation of the mechanism of DNP as a drug for AD treatment should be highly prioritized.

Recently, studies concerning the mechanism of DNP are extremely limited. In 2019, Ghosh et al. revealed the mechanistic pathway of DNP-induced cholinergic inhibition of AD *via* docking and well-tempered metadynamics (WTMtD) stimulations (Duggleby et al., 2019).

## Method

First, the structural formula of donepezil was downloaded from PubChem (https://pubchem.ncbi.nlm.nih.gov/). With the structure acquired, data of the donepezil targeted sites were collected from four separate databases: swisstarget (http://www.swisstargetprediction.ch/), pharmmapper (http://lilab-ecust.cn/pharmmapper/), batman-tcm (http://bionet.ncpsb.org.cn/batman-tcm/), and stitch (http://stitch.embl.de/), each with a different standard for data selection. For swisstarget, data with Probability^*^>0 was included. For batman-tcm, the inclusion standard was set Score cutoff≥ 20. No standard was set for screening in stitch or pharmmapper. The corresponding genes of the donepezil target sites were collected through Uniprot (http://www.uniprot.org/www.uniprot.org/), utilizing data downloaded in pharmmapper.

Three databases were utilized in the searching of pathogenic genes of AD. In GeneCards, genes with a relevance score ≥15 were enrolled. In OMIM, 142 AD-associated genes were included with no screening conducted. In disgenet, genes with score gda ≥0.2 were included. Afterward, data from the three distinct databases were merged and had duplicates removed, leaving 819 distinct genes related to the pathogenesis of AD for subsequent research.

With the screened disease targets, intersections were taken and Venn diagrams were drawn. Four hundred and fourty nine drug targets, 819 disease targets, and 110 intersection targets were obtained. All duplications were removed.

The STRING database was used to construct a disease-drug-active ingredient-key target network, with the minimum required interaction score set as 0.4 and the isolated vertices removed. A target protein interaction network was constructed using Cytoscape3.7.2 software.

With ALB as the brightest in the string hires image and the highest in the string interaction chart, intersections related to ALB are therefore presumed to be of the highest importance.

The intersections of the core genes were collected using the R package with PPI data inputted.

Genes for potential target sites were selected using the R package from the “org.Hs.eg.db” database, and GO enrichment analysis was conducted using DOSE, clusterProfiler, and pathview package (Bioconductor) from three aspects, Biological Process (BP), Cellular Component (CC), and Molecular Function (MF), with p-value cutoff and q-value cutoff both as 0.05. The results from each aspect were visualized with a graph and bubble chart.

With the selected genes, KEGG pathway enrichment analysis was conducted to predict its mechanism of action using DOSE, clusterProfiler, and pathview packages (Bioconductor), with p-value cutoff and q-value cutoff both as 0.05. Columns and bubble charts were generated to visualize the result.

## Results

### Data Processing of Donepezil

**Figure 1:**
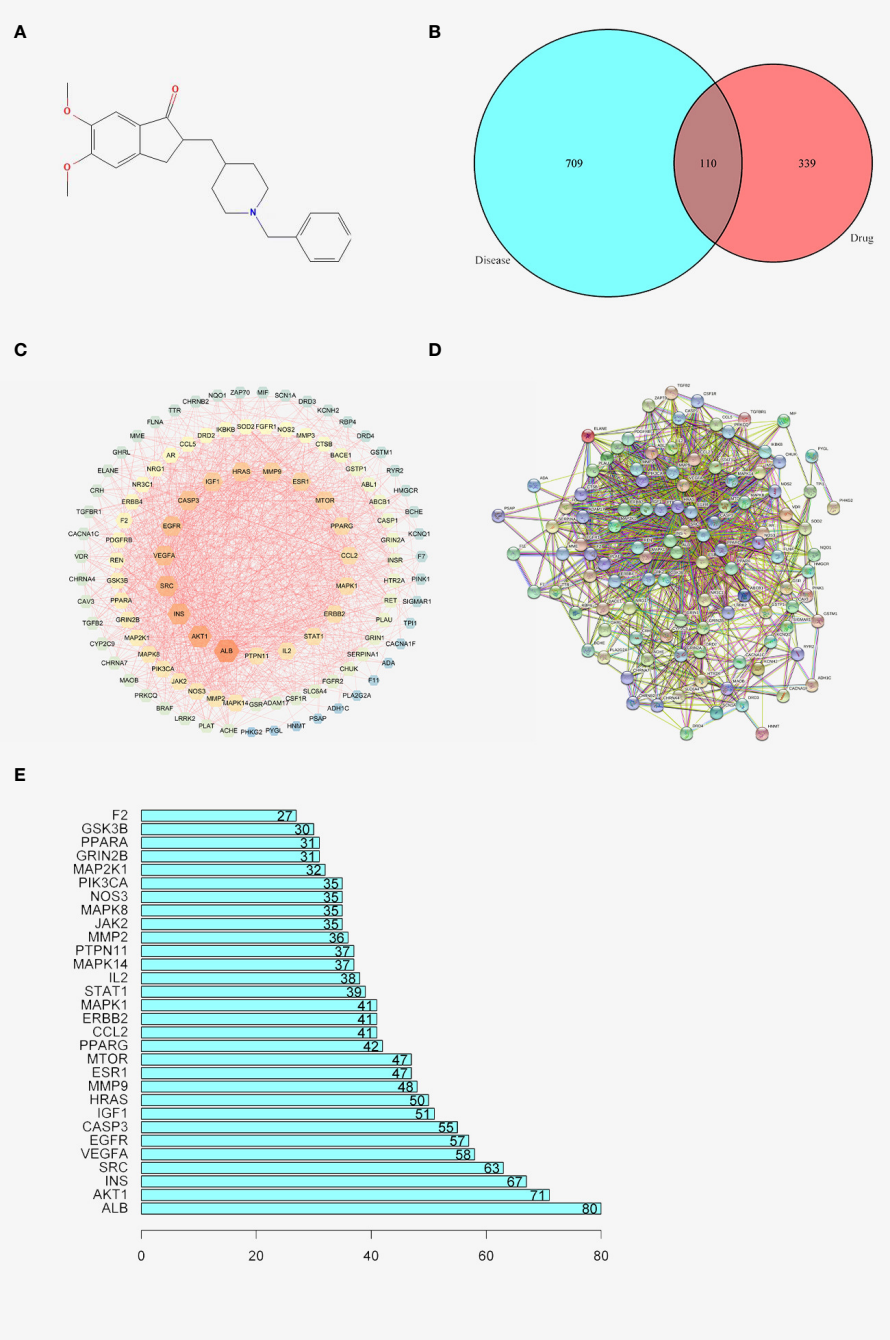
**(A)** 2D structure of DNP. **(B)** Venn graph of AD and DNP. **(C)** Diseases- drug-active ingredient-key target network. **(D)** PPI network. **(E)** Bar chart of 30 core genes.

To acquire the structure of donepezil, the associating structure with Probability^*^>0, score cutoff≥20 was screened and downloaded from the swisstarget and batman-tcm databases. The structure was also searched in the stitch and pharmmapper databases to further verify the results. The structure was visualized using PubChem (Figure 1A) With the selected structure, the associating genes were screened in the Uniprot database (Table 1).

**Table 1:** DNP associated genes.

BCHE ACHE KCNH2 HRH3 SIGMAR1 BACE1 DRD2 DRD4 HTR2A HTR2C MAPK3 MAPK1 OPRD1 OPRK1 CHEK1 MTOR PIK3CA OPRM1 MAP3K12 FGFR1 JAK3 JAK2 SCARB1 MAPK1 MAPK14 MMP2 RET ABCB1 ADRA1A GNRHR SMO AXL CHRM2 GSK3B SYK ERBB2 PAK4 BRAF PTPN11 AURKB STAT1 CHUK DRD3 RPS6KA2 HSD11B1 FLT1 LRRK2 CDK9 ADK PARP1 ABL1 KIT PDE11A JAK1 CDK4 HTR7 PLK1 PIK3CD EGLN1 CCR3 CDK2 TGFBR1 GRIN2A BDKRB1 MELK NPY1R CSF1R POLR1A FLT3 CCR4 CYP24A1 VEGFA MMP9 IGF1 PPIG CCL2 KCNB1 MMP3 ROCK1 AKR1C3 ESR1 MAPKAPK2 PIM1 PPIA PPARG CA2 CFB NR1H2 MAPK8 MAPK10 F2 ALB STS APOA2 NOS3 TTR CASP3 KIF11 EGFR MMP13 PGR AR PDE4D SRC CCNA2 ADAM17 P CACNB1 CACNA1D SCN1A CACNB3 CACNA1F ADRA1B ADRA2C ADRA2A KCNK2 CACNB2 CACNA1S ADRB1 CACNB4 ADRA1D ADRA2B SCN2B GPD1L IL2 GNB5 AIF1 FEZF2 MC2R MAP2K1 RAB3B SCN3B KCNE5 SMAD7 INS SCN4B CRH ARX FLNA CNTN2 DTNBP1 CAV3 KCNA5 PHKG2 ANK2 APLP1 CRHR1 NPPA MC5R PPP1R9B MC3R VAMP2 YWHAE FOXL2 DRD5 OPRL1 RAB3A MC1R ADCY5 AKT1 HRAS MC4R RWDD3 CAV2 ARRDC3 SYT1 DRD1 CABP1 RYR2 ADA PARK2 LHX6 EGR1 DMTN GRIN2B ADORA1 FLOT1 CALCA PINK1 KCNQ1 CASQ2 PALM GJA5 GHRL SLC18A1 PPARA

### Data Processing of AD

A total of 709, 142, and 154 associating genes are screened from the GeneCards, omim, and disgenet database, with the relevance score≥15 and score gda≥0.2. A final number of 819 genes are included after the duplicates were removed (Table 2). Among these genes, 449 genes are related to the drug donepezil, 819 are related to AD, and 110 intersection genes are noted. All the above research into the genes was conducted with the duplicates removed. Venny2.1 (https://bioinfogp.cnb.csic.es/tools/venny/) was utilized to visualize the intersection result (Figure 1B and Table 3).

**Table 2:** Genes related to AD.

PSEN1 APP APOE PSEN2 MAPT SNCA GBA HFE NOS3 PKD1 MT-ND1 PRNP MPO NPC1 LRRK2 TNF IGF1 IL6 IL2 GAA IL10 LMNA PRKN A2M AD5 PLAU ABCA7 MIR146A NOD2 CASP3 SMPD1 VCP AD6 AD10 MIR34A AD7 TGFB1 NEFL MFN2 ACE MIR29A AD11 AD8 AD12 VEGFA AD13 AD14 AD17 TP53 ATP7B MIR106B PARK7 ABCA1 TREM2 GDAP1 ABCA4 ADAM10 IL1B UNC5C MIR107 MTHFR PINK1 SRC ESR1 PSAP DNM2 MTOR GFAP MIR328 PMP22 AGL GDNF NPC2 BDNF GBE1 RYR1 SOD1 APBB1 HTT IFNG GARS1 PIK3CA CYBB CRP TTR MIR298 G6PC1 PTEN UCHL1 FIG4 BACE1 APOA1 GRN ALB SLC17A5 FAS INS CCL2 PON1 NAGLU MAP2K1 CTNNB1 CTSD CFTR EDNRB EGR2 SYNJ1 LRSAM1 ERBB2 SNCB CHAT TLR4 PPARG NDRG1 POLG HSD17B10 TLR2 HSPB1 EGFR KIF1B CXCL8 CYBA AKT1 APOB ACHE LAMP2 IL1A NCF2 TSC2 MIR21 IL4 MAPK1 RAB7A CLU PLP1 COMT GANAB SNCAIP LIPA GSK3B COL4A1 SOX10 LRP1 STAT3 SLC30A4 GAPDHS NFE2L2 PYY HRAS MIR505 NGB MIR766 ADAMTS1 PTGS1 MAPK1 MMP2 DNM1 APLP2 MAPK14 HSF1 IL33 CIB1 IKBKB STAT1 SERPINF1 MT2A BCL2L2 INS-IGF2 ADAM9 CASP12 FERMT2 PTK2B PLCB1 PPARA GRIN2B JAK2 PTPN11

**Table 3:** Intersection genes between AD and DNP related genes.

BCHE ACHE KCNH2 SIGMAR1 BACE1 DRD2 DRD4 HTR2A MAPK1 MTOR PIK3CA SLC6A4 PDGFRB FGFR1 JAK2 PRKCQ RET ABCB1 ERBB2 BRAF IKBKB CHUK DRD3 LRRK2 ABL1 PDE11A TGFBR1 GRIN2A CSF1R VEGFA MMP9 IGF1 CCL2 MMP3 ESR1 MAPK8 F2 ALB TTR CASP3 AR SRC ADAM17 MAPK14 NOS3 NQO1 PPARG MIF ADH1C ERBB4 PTPN11 GSK3B PLAU F7 FGFR2 PLAT GSTP1 PPARA CYP2C9 REN SOD2 MAOB RBP4 MMP2 SERPINA1 ELANE EGFR PLA2G2A TPI1 MME VDR PSAP ZAP70 IL2 HMGCR HNMT F11 NR3C1 PYGL TGFB2 STAT1 CCL5 MAP2K1 CTSB GSTM1 NOS2 CASP1 GSR INSR AKT1 HRAS CHRNB2 GRIN2B CHRNA4 CHRNA7 GRIN1 CACNA1C NRG1 SCN1A CACNA1F INS CRH FLNA CAV3 PHKG2 RYR2 ADA PINK1 KCNQ1 GHRL

### Construction and Analysis of Disease-Drug-Active Ingredient-Key Target Network

The STRING database was used to construct a disease-drug-active ingredient-key target network, with the minimum required interaction score set as 0.4 and the isolated vertices removed (Figure 1C). The brightness, the bigger the dots are, the higher the degree is, representing the importance of the spot to the network.

### Construction and Analysis of PPI Network

To further investigate the mechanism of donepezil's therapeutic effect on AD, 110 intersecting genes were enrolled into the STRING database for PPI network analysis. One hundred and nine core genes and their interacting lines were screened and visualized (Figure 1D and Table 4). The intersections of the core genes were collected using the R package with inputted PPI data. Thirty core genes were finally screened and visualized (Figure 1E).

**Table 4:** 109 core genes and their interaction.

**Order**	**Gene**	**Association**
1	ALB	80
2	AKT1	71
3	INS	67
4	SRC	63
5	VEGFA	58
6	EGFR	57
7	CASP3	55
8	IGF1	51
9	HRAS	50
10	MMP9	48
11	ESR1	47
12	MTOR	47
13	PPARG	42
14	CCL2	41
15	ERBB2	41
16	MAPK1	41
17	STAT1	39
18	IL2	38
19	MAPK14	37
20	PTPN11	37
21	MMP2	36
22	JAK2	35
23	MAPK8	35
24	NOS3	35
25	PIK3CA	35
26	MAP2K1	32
27	GRIN2B	31
28	PPARA	31
29	GSK3B	30
30	F2	27

### GO and KEGG Enrichment Analysis

**Figure 2:**
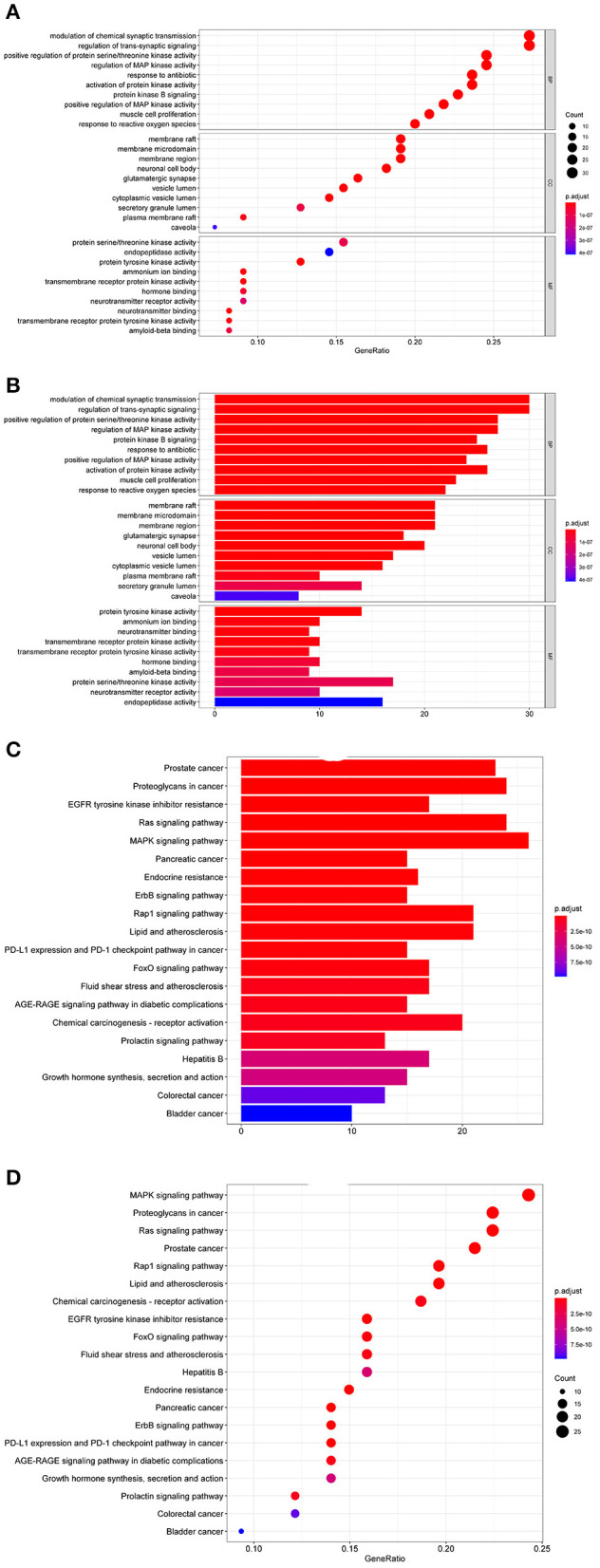
**(A)** Bubble graph of GO enrichment analysis. **(B)** Bar chart of GO enrichment analysis. **(C)** Bar chart of KEGG enrichment analysis. **(D)** Bubble graph of KEGG enrichment analysis.

Genes for potential target sites were selected using the R package from “org.Hs.eg.db” database, and GO enrichment analysis was conducted using the DOSE, clusterProfiler, and pathview packages (Bioconductor) from three aspects, Biological Process (BP), Cellular Component (CC), and Molecular Function (MF), with p-value cutoff and q-value cutoff both as 0.05. The results from each aspect were visualized with a bubble and bar chart (Figures 2A,B). 30 GO lists are acquired (*P* < 0.05, FDR < 0.05), including 10 BP, 10CC, and 10 MF. Therefore, it can be concluded that the mechanism of the therapeutic effect of donepezill to AD could be associated with biological processes such as positive regulation of RNA polymerase II promoter, signal transduction, and positive regulation of DNA template transcription. At the same time, a variety of substances such as plasma membrane, cytosol, and extracellular space are involved, such as protein binding, enzyme binding, transcription factor activity, sequence-specific DNA binding, same protein binding, and other molecular functions.

In KEGG pathway enrichment analysis, 155 signaling pathways are screened (*P* < 0.05, FDR < 0.05), and the top 20 are listed by Count number (Figure 2C). The KEGG analysis indicates that the genes are especially enriched in the MAPK signaling pathway, Ras signaling pathway, and Proteoglycan in cancer. An advanced bubble graph is formed based on the above results, with the signaling pathways as the ordinate and the gene ratio as the abscissa. The count value represents the number of targets enriched on the signal path. The larger the bubble, the more targets enriched, and the color of the bubble represents the p-value (Figure 2D).

## Discussion

The development of drugs with clinical value has met with extreme obstacles (Becker et al., 2008). Over the past 50 years, only two types of drugs are approved for the treatment of AD, which are cholinesterase inhibitors and memantine. Approximately 200 drugs were advanced to at least phase 2 development. However, the therapeutic effects of the approved drugs are limited and there have been disputes on whether they are clinically useful (Schneider et al., 2014).

DNP is a kind of AChEI, which functions as an inhibitor to the cholinesterase enzyme and sustains the activity of acetylcholine at cholinergic synapses. DNP has been shown to significantly improve cognition and daily function and some behavior manifestations of patients with AD (Massoud and Gauthier, 2010). In this study, DNP was taken as the research object. By using the methods of network pharmacology and molecular docking, through the establishment of a disease-drug-active ingredient-key target network, the possible targets and signaling pathways in the treatment of AD were analyzed. The results revealed that the mechanism of its therapeutic effect is multi-component, multi-target, and multi-pathway, laying a foundation for the follow-up in-depth study of the mechanism of DNP in the treatment of AD.

The study received 110 potential targeting genes and 155 signaling pathways in total. The diversity of the associated genes and pathways demonstrate that the mechanism of DNP in the treatment of AD is multifactorial. A strong association between DNP and modulation of chemical synaptic transmission and the regulation of trans-synaptic signaling is noted, further confirming the ability of DNP in sustaining the activity of synapses. In addition, signaling pathway related to the proliferation, differentiation, and survival of cells are also found positively relative, such as Ras and MAPK signaling pathways, suggesting a potential of promoting the growth and proliferation of neurons using DNP.

The cholinergic system plays an indispensable role in neuronal function in memory, learning, and other essential aspects of cognition and plays a wider role in the promotion of neuronal plasticity (Drachman and Leavitt, 1974). Human studies of AD have shown that lesion of the cholinergic system, emerging as early as asymptomatic or prodromal stages of the disease, is one of the most important factors (Schroder et al., 1991). It has been discovered that promotion of cholinergic function in patients with AD may also have durable beneficial biological effects on the brain besides a temporary augmentation of cognitive function (Cavedo et al., 2016, 2017). Cholinesterase inhibitors are designed to inhibit the acetylcholine breakdown and sustain its activity at synapses. There has been evidence that DNP can restore cholinergic function by blocking the enzymes that break down acetylcholine (Lovestone and Howard, 1995). DNP belongs to the most currently available FDA-approved cholinesterase inhibitors for the treatment of AD (Hogan, 2007; Massoud and Gauthier, 2010). Since the number of patients with AD has been on the rise in recent decades, and is becoming a severe social concern, the need to develop an effective drug or therapeutic management for AD is urgent. It is indicated that the implication of cholinesterase inhibitor significantly reduces the risk for nursing home placement by ~30% annually (Feldman et al., 2009). In future studies, more attention should be paid on the mechanism of the pathogenesis of AD and the mechanism of DNP for AD treatment.

In this study, due to the incomplete information in the database and unclear interaction between DNP and other factors, the prediction results have certain limitations. This study did not verify the possible molecular mechanism of DNP in the treatment of AD syndromes either *in vivo* or *in vitro*, and deficiencies remain noticeable. However, this study can provide new ideas and directions for further exploration of relevant experiments.

## Data Availability Statement

The simulation experiment data used to support the findings of this study are available from the corresponding author upon request.

## Author Contributions

LL and PF prepared the figures. JY and YZ finished the paper. All authors contributed to the article and approved the submitted version.

## Funding

This work was supported in part by the Science and Technology Commission of Shanghai Municipality, General Program, No.20ZR1456400.

## Conflict of Interest

The authors declare that the research was conducted in the absence of any commercial or financial relationships that could be construed as a potential conflict of interest.

## Publisher's Note

All claims expressed in this article are solely those of the authors and do not necessarily represent those of their affiliated organizations, or those of the publisher, the editors and the reviewers. Any product that may be evaluated in this article, or claim that may be made by its manufacturer, is not guaranteed or endorsed by the publisher.
